# Indications, Clinical Impact, and Complications of Critical Care Transesophageal Echocardiography: A Scoping Review

**DOI:** 10.1177/08850666221115348

**Published:** 2022-07-19

**Authors:** Ross Prager, Joshua Bowdridge, Michael Pratte, Jason Cheng, Matthew DF McInnes, Robert Arntfield

**Affiliations:** 1 Division of Critical Care, 6221 Western University, Stn B. London, ON, Canada; 2 Department of Medicine, 6363 University of Ottawa, Ottawa, ON, Canada; 3 Department of Radiology, 6363 University of Ottawa, Ottawa, ON, Canada; 4 Clinical Epidemiology Program, 10055 The Ottawa Hospital Research Institute, Ottawa, ON, Canada

**Keywords:** critical care, echocardiography, resuscitation, cardiac arrest, shock, diagnostic imaging, ultrasound

## Abstract

**Background:**

Critical care transesophageal echocardiography (ccTEE) is an increasingly popular tool used by intensivists to characterize and manage hemodynamics at the bedside. Its usage appears to be driven by expanded diagnostic scope as well as the limitations of transthoracic echocardiography (TTE) – lack of acoustic windows, patient positioning, and competing clinical interests (eg, the need to perform chest compressions). The objectives of this scoping review were to determine the indications, clinical impact, and complications of ccTEE.

**Methods:**

MEDLINE, EMBASE, Cochrane, and six major conferences were searched without a time or language restriction on March 31^st^, 2021. Studies were included if they assessed TEE performed for adult critically ill patients by intensivists, emergency physicians, or anesthesiologists. Intraoperative or post-cardiac surgical TEE studies were excluded. Study demographics, indication for TEE, main results, and complications were extracted in duplicate.

**Results:**

Of the 4403 abstracts screened, 289 studies underwent full-text review, with 108 studies (6739 patients) included. Most studies were retrospective (66%), performed in academic centers (84%), in the intensive care unit (73%), and were observational (55%). The most common indications for ccTEE were hemodynamic instability, trauma, cardiac arrest, respiratory failure, and procedural guidance. Across multiple indications, ccTEE was reported to change the diagnosis in 52% to 78% of patients and change management in 32% to79% patients. During cardiac arrest, ccTEE identified the cause of arrest in 25% to 35% of cases. Complications of ccTEE included two cases of significant gastrointestinal bleeding requiring intervention, but no other major complications (death or esophageal perforation) reported.

**Conclusions:**

The use of ccTEE has been described for the diagnosis and management of a broad range of clinical problems. Overall, ccTEE was commonly reported to offer additional diagnostic yield beyond TTE with a low observed complication rate. Additional high quality ccTEE studies will permit stronger conclusions and a more precise understanding of the trends observed in this scoping review.

## Background

Point-of-care ultrasound (POCUS) has become an important tool for the modern intensivist.^1^ An iconic application of POCUS is the assessment of cardiorespiratory failure where critical care echocardiography (CCE – a sub-domain of POCUS) has been shown to rapidly and accurately inform management of the anatomic, hemodynamic and non-cardiac etiologies of shock and is endorsed as a first-line assessment tool for shock by the European Society of Intensive Care Medicine.^1–6^

While CCE has traditionally been viewed as a transthoracic technique, challenges with patient positioning, limited acoustic windows, and the need to perform other interventions (eg, chest compressions) are common barriers to its use.^7^ In response to these challenges, critical care transesophageal echocardiography (ccTEE) has seen increased adoption.^8,9^ The probe's close proximity to the heart yields reliable, high quality images no matter patient body habitus or positioning. As well, its indwelling nature facilitates serial assessments and the ability to be performed during cardiac arrest without interrupting chest compressions.^10,11–13^ These factors have led the American Society of Echocardiography to state that a primary indication for TEE is the lack of transthoracic windows in a critically ill patient where echo is expected to change management.^9^

ccTEE is a modality of scalable complexity, from goal-directed TEE that uses “core” views to guide resuscitation,^14^ to detailed assessments of hemodynamics and the assessment of extra-cardiac structures in the thorax and abdomen.^15–17^ ccTEE differs from consultative TEE (eg, cardiology performed) in that the ccTEE provider's role in patient care includes the real time integration of findings into diagnosis, hemodynamic management and prognosis.^18^

As the literature supporting ccTEE grows, a synthesis of primary studies is expected to aid in understanding and influencing the adoption of ccTEE among intensivists. In conducting this scoping review, our objectives were to characterize the indications, clinical impact, complications and domains requiring further inquiry for the ccTEE modality.

## Methods

The study protocol and all data are available on Open Science Framework (OSF) (https://osf.io/fsnt3/). Ethics approval for scoping reviews is not required at our institutions. The study adheres to the Preferred Reporting Items for Systematic Reviews and Meta-analyses extension for scoping reviews (PRSIMA-SCR) guidelines.^19^

### Search Strategy

The search strategy was designed with the help of a research librarian (RS) and is summarized in Appendix 1. MEDLINE, EMBASE, and Cochrane Central Register of Controlled Trials were searched without a time or language restriction on March 31^st^, 2021. Clinicaltrials.gov and OSF were also searched for ongoing TEE studies (Appendix 2). Six critical care and emergency medicine conferences had their abstracts searched form 2018 to present (Appendix 3). Any relevant systematic review identified by our search had its references screened. Google Translate (Google, USA) was used for non-English studies with a human translator employed when needed.

### Study Selection

Studies were included if they: (1) assessed adult human subjects (≥ 16 years old); (2) investigated the use of TEE in critically ill patients; (3) the TEE was performed in a critical care context with focused indications (as opposed to comprehensive TEE performed by cardiologists); (4) the TEE was performed in the intensive care unit (ICU), emergency department (ED), or pre-hospital setting (eg, ambulance). Goal directed TEE performed by cardiologists were included.

Studies were excluded if they: (1) investigated comprehensive cardiology performed TEE; (2) investigated intra-operative or post-cardiac surgical TEE; (3) used TEE to determine cardiac dimensions without a direct clinical application; (4) were reviews, opinion pieces, educational studies, or letters to the editors.

Covidence software (Veritas Health Innovation, Australia) was used for abstract and full-text screening. Abstracts and full texts were screened independently and in duplicate by 2 authors (RP and JB) with consensus used for discrepancies. Screening of the conference literature was done in duplicate by RP and MP.

### Data Extraction

The following data was extracted independently and in duplicate by two authors (RP and JB), with discrepancies resolved through consensus discussion: study author, year of publication, country of corresponding author, setting (academic, community, mixed), study type, study design (prospective, retrospective), clinician performing TEE (intensivist, anesthesiologist, emergency physician), training of clinician performing TEE, number of patients, indication for TEE, primary outcome being measured, main finding(s), and TEE-related complications. Two pilot extractions were done by RP and JB.

### Bias Assessment

In keeping with the PRISMA-SCR guidelines, studies were not assessed for applicability, risk of bias, or publication bias.

### Outcomes

The primary questions addressed by this review are: (1) What are the reported indications for ccTEE? (2) What is the reported impact of ccTEE on diagnosis, management, and patient outcomes? The secondary questions addressed by this review are: (1) What are the study demographics? (2) What complications are reported for ccTEE?

### Analyses

No analyses were performed. Descriptive statistics, count data, and percentages were used for the demographic information of the primary studies. All data extraction was done in Microsoft Excel (Washington, USA).

## Results

### Study Demographics

**Figure 1.**
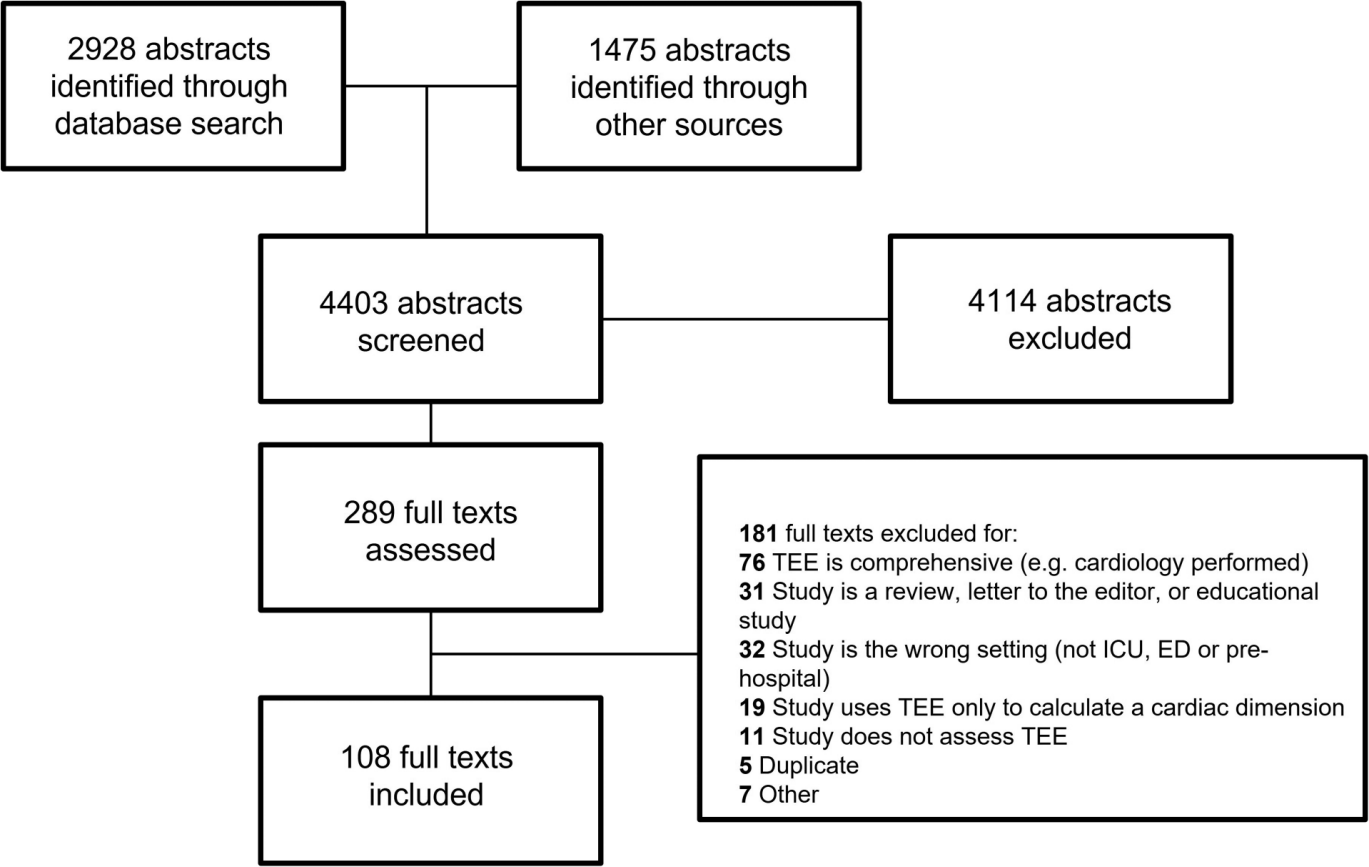
Flow Diagram.

Of the 4403 abstracts screened, 289 studies underwent full-text review, with 108 studies (6739) patients) included (Figure 1). Study demographics are provided in Table 1. Case studies are summarized in Appendix 4. All included studies are listed in Appendix 5.

**Table 1.** Study Demographics.

Demographics	Number of studies n (%)
All Included Studies	108 (100%)
Country of Corresponding Author	
USA	35 (32%)
France	24 (22%)
Canada	8 (7%)
Other	41 (38%)
Location	
ICU	76 (73%)
ED	22 (20%)
Other (Prehospital, Ward, Radiology, PACU)	4 (4%)
Mixed	1 (1%)
Not Reported	5 (5%)
Setting	
Academic	91 (84%)
Community	5 (5%)
Mixed	0
Not Reported	12 (11%)
Study Type	
Case Study or Series	42 (39%)
Observational	59 (55%)
Diagnostic Accuracy	6 (6%)
Randomized Control Trial	1 (1%)
Study Design	
Prospective	36 (33%)
Retrospective	71 (66%)
Not reported	1 (1%)
Clinician Performing Scan	
Intensivist	34 (32%)
Anesthesiologist	8 (7%)
Emergency Physician	13 (12%)
Mixed	1 (1%)
Not reported	52 (48%)
Total Number of Patients who underwent TEE	6739

### ccTEE in Critically Ill Patients

For studies assessing the general use of ccTEE (ie studies not focused on a single indication), the most common indications were to assess hemodynamics (40%-70% of patients),^10,20,21^ to rule out endocarditis (18%-33% of patients),^10,20–22^ and for cardiac arrest (20% of patients) (Table 2).^10^ ccTEE was reported to help change diagnosis in 52% to 78% of cases^23,24^ and resulted in a change in management in 32% to 79% of cases.^10,20,21,23,25,26^ When miniature or disposable TEE probes were compared to standard TEE probes, the miniature probes had similar diagnostic performance for 2 dimensional echocardiography.^27–29^

**Table 2.** Studies That Reported the General Use of ccTEE (ie, Not Limited to One Specific Indication).

Study	Study Type and Design	Clinician, Location, and Training	Primary Outcome(s)	Patients (n)	Main Finding(s)	Compli-cations
Arntfield et al 2020	Retrospective Observational	Intensivist, ICU, Mixed experience	Indications, Change in Management	274	The most common indications for TEE were hemodynamic instability (45.2%), assessment for infective endocarditis (22.2%) and cardiac arrest (20.1%). TEE had a significant impact on management.	None
Bagate et al 2020	Prospective Observational	NR, ICU, NR	Pneumonia following TEE	100	There was no difference in tracheal pepsin or amylase levels (biomarkers of microaspiration) following TEE. There was a high frequency of pneumonia following TEE so a causative mechanism cannot be ruled out.	None
Beyles et al 2020	Retrospective Observational	NR, ICU, NR	Feasibility of TEE in prone patients	17	TEE is feasible in prone patients. Tricuspid Longitudinal Annular Displacement is a potential method to assess for RV function	None
Wray et al 2019	Retrospective Observational	EP, ICU/ED/Prehospital, Varied experience	TEE Related Complications	228	80 high-risk patients with coagulopathy and 148 low-risk patients were assessed for complications related to TEE. In total, 8 (4%) complications were reported, 4 (5%) in the high-risk group and 4 (3%) in the low risk group. No deaths attributed to TEE. Complications were mainly mild UGIB.	8 mild complications (GI bleeding)
Lau et al 2018	Retrospective Observational	Intensivist, ICU, Mixed experience	Correlation between cardiology and intensivist interpreted TEE	56	Compared to TEE read by cardiologists, critical care TEE had high (>90%) sensitivity and specificity for the diagnosis of most cardiac pathology	None
Garcia et al 2017	Retrospective Observational	Intensivist, ICU, 4 h training	Indications, Change in Diagnosis and Management	152	The most common indications for TEE were cardiorespiratory failure (70%) and to rule out endocarditis (18%). TEE resulted in a new diagnosis in 28% of cases and change in management in 38% of cases.	None
Arntfield et al 2015	Retrospective Observational	EP, ED, minimal training (2 h didactic 2 h hands-on)	Change in diagnosis and management	54	The implementation of TEE in the ED for cardiac arrest, post-arrest shock, and hypotension had a diagnostic impact in 78% of cases and a therapeutic impact in 67% of cases.	None
Begot et al 2015	Prospective Observational	Intensivist, ICU, Experienced in TEE	Correlation between miniature and standard TEE probe	57	A miniaturized TEE probe has similar diagnostic performance and high correlation to findings on a standard TEE probe	None
Cioccari et al 2013	Prospective Observational	Intensivists, ICU, 5 h training	Correlation between miniaturized TEE and cardiology performed TEE, change in management	55	There was a high degree of interrater reliability between ccTEE and expert cardiology performed TEE. Of the 148 ccTEE assessments, changes in management were reported after 50 (34%) of ccTEE exams.	None
Dessap et al 2011	Prospective Observational	NR, ICU, NR	Feasibility of TEE in prone patients	34	TEE insertion is feasible in the majority (33/34) of intubated, prone patients in the ICU with excellent views obtained for the majority of patients	None
Orme et al 2009	Retrospective Observational	Intensivist, ICU, Mixed	Indications, Change in Management	217	The most common TEE indications were assessment of LV function (46.1%) and hypotension (16.6%). TEE was reported to change management 53.5% of the cases it was used.	None
Colreavy et al 2002	Retrospective Observational	Intensivist, ICU, Fellowship Trained	Indications, Change in Management	255	The most common indications for TEE were hypotension (40%), to rule out endocarditis (27%), and the assessment of ventricular function (15%). TEE changed management in 32% cases.	None
Benjamin et al 1998	Prospective Observational	Intensivist, ICU, 10 supervised scans	Correlation between intensivist and cardiology interpreted TEE	100	Intensivist interpretation matched the cardiologist interpretation in 93% of cases for LV thickness, 87% for intracardiac volume status, 81% for LV RWMA and 77% for LV function. The TEE data did not correlate well with pulmonary artery catheter data	None
McLean et al 1998	Retrospective Observational	Intensivist, ICU, NR	Indication, Findings	53	The most common indications for TEE were to assess ventricular function (41.5%), assess for cardiac source of embolism (24.5%), and endocarditis (11.3%). Specific abnormalities were found in 34.0% of studies and incidental findings found in 11.3%.	None
Islama et al 1996	Prospective Observational	NR, ICU, NR	Indications, Change in Management	61	The most common indications for TEE were hypoxia, hypotension, and to rule out endocarditis. TEE provided critical information not obtained by TTE 45% of patients.	4 – 2 sedation related hypotension, 1 minor bleed, 1 aspiration
Mentec et al 1995	Prospective Observational	Intensivist, ICU, NR	Bacteremia following TEE	139	2 / 139 (1.4%) of TEE studies resulted in probable bacteremia, with new blood culture positivity not felt to be contamination after TEE was performed. This did not differ whether the patient was receiving antibiotics or not.	2 – possible bacteremia
Vignon et al 1994	Prospective Observational	Intensivist, ICU, NR	Additional diagnostic yield of TEE to TTE	96	TEE had higher diagnostic accuracy (96.9%) compared to TTE (38.0%) across broad indications in the ICU.	None
Chenzbraun et al 1993	Retrospective Observational	NR, ICU, NR	Diagnostic Yield	100	Of the 113 TEE exams on 100 patients, 51 (45.1%) showed findings that were considered significant. Aortic dissection was diagnosed by TEE in 16 patients. 9/33 studies in sepsis for suspected IE were positive. 5/19 positive for suspicion of embolic event. 4/15 determined pathology for hypotension.	7 – minor hypotension in 5, and mild hypoxia in 2

Abbreviations: NR, Not Reported; ICU, Intensive Care Unit; RWMA, Regional Wall Motion Abnormalities; TBPT, Transpulmonary Bubble Transit; VAP, Ventilator Associated Pneumonia; ARDS, Acute Respiratory Distress Syndrome; UGIB, Upper Gastrointestinal Bleeding.

### ccTEE to Investigate Hemodynamic Instability and Shock

In many cases, ccTEE was reported to help clarify the etiology of shock (Table 3). ccTEE was used to correctly identify right ventricular (RV) failure from acute pulmonary embolism,^30–34^ for managing the peripartum patient with hemodynamic collapse,^35,36^ and for identifying unexpected causes of shock like dynamic left ventricular outflow tract (LVOT) obstruction.^37,38^ There were a number of case reports of ccTEE identifying rare causes of shock that may not have been visualized on TTE: right atrial compression from intrabdominal hematoma,^39^ thoracic tamponade post lung transplant,^40^ and regional pericardial tamponade in the ED after discharge home from cardiac surgery.^41^ For patients who already had TTE performed, ccTEE resulted in additional changes in management in 40% of cases, and refuted TTE diagnoses in approximately 20% of cases (Table 3).^42,43^

**Table 3.** ccTEE for Shock and Hypotension.

Study	Study Type and Design	Clinician, Location, and Training	Primary Outcome(s)	Patients (n)	Main Finding(s)	Compli-cations
Si et al 2020	Prospective Observational	Intensivist, ICU, NR	Additional diagnostic yield compared to TTE	68	TEE provided additional yield compared to TTE in 62% of patients, particularly for those with inadequate TTE windows. This resulted in important changes in management for 46% of patients.	None
Merz et al 2019	Prospective RCT	Intensivist, ICU, Experienced Clinicians	Time to resolution of shock	271	Patients were randomized to hTEE with disposable TEE that could be left in situ for 72 h versus standard care. There was no difference in the primary outcome of time to resolution of hemodynamic instability at 6 days. Secondary outcomes of number of patients with hemodynamic resolution at 72 h, as well as the per protocol analysis of time to resolution of hemodynamic instability was shorter in the hTEE group.	1 – minor oropharyngeal bleeding
Younan et al 2019	Retrospective Observational	Intensivist, ICU, NR	Association between LVEI and AKI	132	Measured LV eccentricity index (LVEI), a measure of RV volume loading in diastole, in patients who underwent TEE showed a significant inverse association with AKI (odds ratio 0.03, 95% confidence interval 0.00-0.68).	None
Griffin et al 2017	Retrospective Observational	NR, ICU, NR	Need for continuous Renal Replacement Therapy (CRRT)	13	Pre and post study where 23 patients in the pre-TEE cohort who required CRRT were compared with 13 patients in the TEE cohort who required CRRT. There was a decrease in the percentage of patients requiring CRRT after implementation of TEE (OR 2.5) *P* = .014.	None
Hlaing et al 2017	Prospective Observational	Intensivist, ICU, Extensive Experience with TEE	Change in management	53	TEE obtained clinically meaningful information in 77% of patients it was used	None
Vignon et al 2016	Prospective Diagnostic Accuracy	Intensivist, ICU, Advanced echocardiography	Prognostic Accuracy to predict fluid responsiveness	540	SVC variation with a cut-off of 21% had a sensitivity of 61% and specificity of 85% for predicting fluid responsiveness in mechanically ventilated patients. This was superior to pulse pressure variation and IVC respiro-phasic variation.	None
Burrage et al 2015	Retrospective Observational	Anesthesiologist, NR, Board Certified	Pathology detected on TEE	10	For hemodynamically unstable obstetrical patients, TEE was helpful to identify the etiology of shock and to guide cardiac arrest management. TEE identified intracardiac thrombus, hypovolemic shock, right heart dysfunction, and a hemodynamically important pericardial effusion.	None
Fletcher et al 2015	Retrospective Observational	Intensivist, ICU, Varied	Change in management	41	TEE led to change in management in 90.2% patients it was implemented in.	2 patients: lip ulcer, mild bleeding
Charbonneau et al 2014	Prospective Diagnostic Accuracy	NR, ICU, Experienced Clinicians	Prognostic Accuracy to predict fluid responsiveness	44	SVC collapsibility index of >36% had an auROC of 0.74 for predicting fluid responsiveness in mechanically ventilated patients. The optimal threshold for SVC collapsibility index was 29% which yield a sensitivity of 54% and specificity of 89%. SVC collapsibility was significantly more accurate for predicting fluid responsiveness than IVC.	None
Verma et al 2009	Prospective Observational	NR, ICU, NR	Diagnostic Yield	12	For patients with inadequate TTE views, TEE identified pathology in 58% of cases	None
Schneider et al 2007	Prospective Observational	Anesthesia, ICU, Experienced (>74 scans/year)	Comparison of handheld versus standard TEE image quality	18	Handheld TEE was compared to standard TEE. The time and image quality were graded as equal for most scans except for valvular color doppler imaging which was superior on the standard TEE.	None
Brederlau et al 2006	Prospective Observational	Anesthesiologist, ICU, Experienced (>100 TEE)	Findings, Change in Management	339	For hemodynamically unstable patients, the most common TEE findings were volume depletion (47%), regional wall motion abnormalities (27%) and global left ventricular dysfunction (22%). TEE changed the management plan in 31% of cases. Of the TEE studies, 56 provided additional information with therapeutic relevance in 45% cases.	None
Veillard-Baron et al 2006	Prospective Observational	Intensivist, ICU, experienced (>60 TEE)	Correlation of qualitative with quantitative measurements	30	Qualitative measurement of SVC variation, LV function, and RV function correlate highly with quantitative measurements and may be sufficient for goal directed echocardiography	None
Denault et al 2002	Prospective Observational	Anaesthesiologist, ICU, NR	Change in management	214	TEE was reported to change management for 40% of patients.	None
Feissel et al 2001	Prospective Observational	NR, ICU, NR	Prognostic Accuracy	19	Peak Velocity variation in aortic velocity on TEE (on a beat-to-beat basis) was higher in fluid responsive compared to non-fluid responsive patients with septic shock, with a threshold of 12% yielding a sensitivity of 100% and specificity of 89%.	None
Krivec et al 1997	Prospective Diagnostic Accuracy	NR, ICU, NR	Diagnostic Accuracy	24	TEE had high sensitivity (92%) and specificity (100%) for the diagnosis of massive PE in the presence of RV failure	None
Sohn et al 1995	Retrospective Observational	NR, ICU, Not stated	Indications, Diagnostic Yield	124	Common indications for TEE included assessment of LV function, assessment of valvular function, ruling out endocarditis, and assessing for tamponade. It identified a cause of hemodynamic instability in 52% of patients	None

Abbreviations: CRRT, Continuous renal replacement therapy; SVC, Superior vena cava; LV, Left Ventricle; RV, Right Ventricle.

**Table 4.** ccTEE use in Cardiac Arrest.

Study	Study Type and Design	Clinician, Location, and Training	Primary Outcome(s)	Patients (n)	Main Finding(s)	Compli-cations
Jung et al 2020	Retrospective Observational	EP, ED, 10 proctored TEE	Pathology identified on TEE	158	For patients with OHCA, TEE identified pathology in 25% of cases. Common pathologies were aortic dissection (48%), PE (20%), and intracardiac thrombi (23%).	None
Kim et al 2020	Retrospective Observational	EP, ED, 2 h didactic, 10 proctored scans	Detection of aortic dissection	45	For patients with OHCA, 22% patients were diagnosed as having aortic dissection based on TEE, of which, none survived. Other findings included 5 PEs (11%) and 2 isolated tamponade (4%).	None
Catena et al 2019	Retrospective Observational	Intensivist, ED, “Expert in Echocardiography”	TEE findings associated with ROSC	19	LVOT opening during CPR was present in all patients with ROSC (7/7, 100%) compared to (1/12, 8%) patient who did not survive *P* = .0002	None
Fair et al 2019	Retrospective Observational	EP, ED, “Additional Training”	Pulse Check Duration	25	For patients with OHCA, TEE was associated with shorter CPR pulse check duration (9s [5-12s]) compared to TTE (19s [16-22s]) or manual (11s [8-14s])	None
Teran et al 2019	Prospective Observational	EP, ED, 10 supervised high fidelity scans	Feasibility, Change in Management	33	For patients with OHCA, TEE resulted in the change in management in 97% of cases. Management changes included optimization of CPR position, defibrillating fine ventricular fibrillation not seen on ECG, discontinuing resuscitation effort due to no cardiac motion, and identification of cardiogenic shock post ROSC with initiation of VA-ECMO.	None
Hwang et al 2009	Prospective Observational	NR, ED, NR	Area of maximal compression (AMC) during CPR	34	During CPR, an AMC over the left ventricle highly correlates with increased stroke volume. Many patients will have the AMC on or near the LVOT which impairs CPR quality.	None
Van der Wouw et al1997	Prospective Diagnostic Accuracy	NR, ED, NR	Diagnostic Accuracy for the etiology of CA	48	Compared to autopsy, clinical follow-up, or surgical findings, TEE had a sensitivity of 93% and specificity of 50% for determining the etiology of cardiac arrest. Common etiologies included MI, PE, and aortic dissection	None

Abbreviations: EP, Emergency Physician; ED, Emergency Department; OHCA, Out of hospital cardiac arrest; ROSC, Return of Spontaneous Circulation; TEE, Transesophageal Echocardiography; CPR, Cardiopulmonary Resuscitation; TTE, Transthoracic echocardiography; VA-ECMO, Veno-arterial Extracorporeal Membrane Oxygenation; LVOT, Left Ventricular Outflow Tract; NR, Not reported; CA, Cardiac Arrest; MI, Myocardial Infarct; PE, Pulmonary Embolism.

For patients in shock, ccTEE was also able to predict fluid responsiveness.^44–46^ Superior vena cava (SVC) collapsibility assessment performed using TEE had moderate diagnostic accuracy to predict fluid responsiveness, with thresholds for collapsibility of 21% to 29% yielding a sensitivity of 54% to 61% and specificity of 85% to 89%.^44,45^ ccTEE also identified ventricular failure that resulted in the initiation or titration of vasopressors and inotropes in 4% to 73% of cases.^43,47,48^

Although most ccTEE studies are observational, a randomized controlled trial (n = 550) was performed to determine whether a disposable TEE (hTEE) impacted time to resolution of hemodynamic instability for critically ill patients. Although there was no difference in the intention-to-treat (ITT) analysis of the primary outcome of resolution of hemodynamic instability at 6 days (sub-hazard ratio (SHR) 1.20, 95% confidence interval (CI) 0.98-1.46), there was a reduction in time to resolution of hemodynamic stability within 72 h of placement of the hTEE probe (SHR 1.26, 95% CI 1.02-1.55).^49^

### ccTEE use in Cardiac Arrest

ccTEE in cardiac arrest identified the etiology of cardiac arrest in 25% to 35% of cases,^50–53^ and in one study was reported to change management in 97% of cases in the intra or peri-arrest period.^52^ Multiple studies reported that ccTEE identified fine ventricular fibrillation (VF) not seen on surface electrodes.^32,36,52^ In one study, ccTEE detected pseudo-pulseless electrical activity (PEA) in 28% of cases of PEA, where ventricular contraction occurred but did not generate a palpable pulse.^52^ Intra-arrest TEE was reported to improve CPR quality through guiding chest compressions to avoid ineffective compressions over the LVOT or aortic root.^11,23,52^ Proper compression location over the LV was associated with increased stroke volume generated by chest compressions, and potentially improved survival.^13,52,54^ ccTEE was also associated with shorter pulse checks when compared to both TTE and manual pulse checks.^11^ Using protocols that focus on core TEE views, ccTEE in cardiac arrest was feasible and safe for providers with limited training.^11,50–52,55^ A summary of the use of ccTEE in cardiac arrest is provided in Table 4.

### Procedural Guidance with ccTEE

ccTEE was also used to help guide invasive procedures, including confirming guidewire placement during the initiation of veno-venous (VV) and veno-arterial (VA) extracorporeal membrane oxygenation (ECMO) (Table 5).^40,52,55–59^ Once ECMO was established, ccTEE was used to troubleshoot flow issues and guide cannula repositioning, which was required in up to 38% of patients in one VV-ECMO cohort.^59^ In one small cohort of patients on VA-ECMO, TEE guided weaning protocols performed by intensivists had high predictive accuracy (100%) for successful weaning.^60^ TEE was also used to provide real-time guidance for the insertion of transvenous pacemakers.^36,61,62^

**Table 5.** TEE Guided Procedures.

Study	Study Type and Design	Clinician, Location, and Training	Primary Outcome(s)	Patients (n)	Main Finding(s)	Compli-cations
Griffee et al 2020	Retrospective Observational	Anesthesiologist, ICU, NR	TEE guided VV-ECMO cannula placement	42	TEE guided VV-ECMO cannulation resulted in 87% patients having correct cannula position. 38% of patients required TEE guided cannula adjustments during the course of their ECMO run.	None
Fair et al 2016	Retrospective Observational	EP, ED, 10 supervised TEE	Successful TEE guidance of ECMO cannulation	10	For patients with OHCA, TEE was used to guide VA-ECMO cannulation for 10 patients in cardiac arrest. TEE was used to confirm guidewire placement in the artery and vein, as well as for positioning the venous cannula.	None
Nowak-Machen et al 2016	Retrospective Observational	Anesthesiologist, ICU, Extensive Experience with TEE	TEE related complication	53	For patients receiving ECMO who underwent TEE, 2/53 (4%) of patients developed oropharyngeal bleeding that did not require intervention.	2 - minor
Cavarocchi et al 2013	Prospective Observational	Intensivist, ICU, NR	Success at weaning VA-ECMO	21	For patients with cardiogenic shock, a standardized VA-ECMO weaning protocol using a disposable indwelling TEE probe had 100% accuracy for determining successful weaning from VA-ECMO	None

Abbreviations: OHCA, Out of hospital cardiac arrest; TEE, transesophageal echocardiography; VA-ECMO, Veno-arterial extracorporeal membrane oxygenation.

### ccTEE use in Trauma

TEE use in trauma had high diagnostic accuracy (>90%) for the detection of traumatic aortic injuries.^63–65^ After initial surgical hemostasis, TEE helped identify other etiologies of shock including LV and RV dysfunction, blunt cardiac injury, and traumatic valvular abnormalities.^66,67^ TEE was also described during the management of critically ill burn patients, with its use associated with a change in diagnosis and management in several small studies.^68–71^ The use of TEE in trauma is summarized in Table 6.

**Table 6.** TEE use in Trauma.

Study	Study Type and Design	Clinician, Location, and Training	Primary Outcome(s)	Patients (n)	Main Finding(s)	Compli-cations
Younan et al 2018	Retrospective Observational	Intensivist, ICU, NR	Association between RV dysfunction and outcome	74	Reduced RV Fractional Area of Change was associated with longer days of mechanical ventilation in burn patients.	None
Held et al 2016	Retrospective Observational	Intensivist, ICU, minimal training (8 h didactic, 2 h in person)	Feasibility	11	Even with minimal training, TEE can be used to guide resuscitation in burn patients. Its use identified LV and RV dysfunction, initiate inotropes, and guide fluid resuscitation.	None
Metaxa et al 2011	Retrospective Observational	Intensivist, ICU, Echo certified	Identification of endograft malposition	14	TEE correctly identified 29% of cases of aortic endograft malposition following endovascular repair of the aorta from traumatic aortic injury	None
Etherington et al 2010	Retrospective Observational	Anesthesiologist, ICU, TEE Board Certified	Indications, Change in Management	17	In a burn ICU, the main indications for TEE included hypotension and to rule out endocarditis. In 24% of patients the TEE findings changed management	None
Burns et al 2005	Retrospective Observational	Anesthesiologist, ICU, TEE Board Certified	Change in Management	25	For critically ill trauma patients with ongoing shock despite hemorrhage control, 64% of patients had TEE findings that changed management plans.	None
Huttemann et al 2002	Retrospective Observational	NR, ICU, NR	Presence of LV dysfunction	51	For severe lethal brain injured patients, TEE identified LV dysfunction in 14% of patients.	None
Smith et al 1995	Prospective Diagnostic Accuracy	NR, ED, NR	Diagnostic Accuracy for TDA	101	TEE had a sensitivity of 100% and specificity of 98% (1 false positive) for the diagnosis of TDA compared to aortography, surgery, or autopsy as the reference standard	None
Vignon et al 1995	Prospective Diagnostic Accuracy	NR, ED, NR	Diagnostic Accuracy for Traumatic Aortic Disruption (TDA)	32	Compared to necropsy, surgery or autopsy as the reference standard, TEE was 91% sensitive and 100% specific for TDA	None
Vignon et al 1998	Retrospective Diagnostic Accuracy	NR, NR, NR	Diagnostic Accuracy for Great Vessel Injury	41	TEE has moderate sensitivity (80%) and specificity (92%) for detecting hemomediastinum, which may be indicative of major vascular injury	None

Abbreviations: NR, Not reported; ED, Emergency Department; ICU, Intensive Care Unit; LV, Left Ventricle; RV, Right Ventricle.

### TEE use in Respiratory Failure

Several studies have described the use of transesophageal lung ultrasound (TELUS), which is effective at identifying basal and posterior lung pathology.^72,73^ Additionally, ccTEE use in patients with ARDS can identify RV failure and intracardiac shunting.^74–77^ TEE use in respiratory failure is summarized in Appendix 6 and 7.

### TEE-Related Complications

There were two significant gastrointestinal bleeds reported as a complication of ccTEE: one requiring endoscopic intervention, and the other requiring transfusion for hypovolemia.^78^ There were no other major ccTEE associated complications (death or esophageal perforation) reported. There were 29 minor complications reported in 6739 patients, with those studies reporting a 1.6% to 9.8% minor complication rate (Table 7). One study identified 2 cases of bacteremia that they felt were likely associated with TEE use.^79^ Another showed TEE was not associated with biomarkers of aspiration.^80^

**Table 7.** Summary of ccTEE Related Complications.

Study	Indication for TEE	Experience of Providers	Number of Patients	Complication	Number of complications (Major/Minor)
Marchandot et al 2018	Cardiac Arrest	NR	1	Left atrial intramural hematoma complicating CPR while TEE in situ for an elderly patient on warfarin. No intervention required.	0 Major/ 1 Minor
Wray et al 2019	General	Multiple levels (well defined): resuscitative, basic, and comprehensive	228	2 significant upper gastrointestinal bleeds: 1 requiring transfusion, 1 requiring endoscopy 3 mild upper gastrointestinal bleed 1 vocal cord paralysis listed as possibly related to TEE	2 Major Bleeds / 4 Minor
Colreavy et al 2002	General	Experienced with 6 months of TEE fellowship	255	2 Transient hypotension 1 Oropharyngeal bleeding 1 Peri-intubation aspiration	0 Major / 4 Minor
Vieillard-Baron et al 2013	Hemodynamic	Experienced	94	2 mild gastric bleeding 2 lip ulceration	0 Major / 4 Minor
Merz et al 2019	Hemodynamic	Experienced Intensivists	271	1 oropharyngeal bleeding	0 Major / 1 Minor
Slama et al 1996	General	NR	61	1 spontaneously resolving atrial fibrillation	0 Major / 1 Minor
Nowak-Machen et al 2016	Procedural (ECMO)	Extensive Experience with TEE	53	2 oropharyngeal bleeding	0 Major / 1 Minor
Fletcher et al 2015	Hemodynamic	Varied (5 senior, consultant- level cardiac intensivists certified in TEE and 4 senior trainees who had completed a 3-day introductory course on basic TEE)	41	1 lip ulcer 1 bleeding suctioned through nasogastric tube 1 inadvertent probe removal 1 probe requiring removal due to disruption of electrical conductor	0 Major / 4 Minor
Chenzbraun et al 1994	General	NR	100	5 transient hypotension 2 transient hypoxemia	0 Major / 7 Minor

## Discussion

The range of applications for ccTEE is broad, from resuscitative views acquired during cardiac arrest to advanced applications that mimic the complexity of consultative TEE.^18^ Given the relative infancy of ccTEE, it's evidentiary base consists largely of case studies and retrospective observational studies, however, there are a number of well-designed prospective studies advancing the field. The existing ccTEE literature has characterized the indications for ccTEE and highlighted cases of additional diagnostic yield compared to TTE but has not consistently demonstrated improvement in patient important outcomes from the integration of ccTEE into existing diagnostic pathways. This is not surprising given the number of steps required for a diagnostic test to improve outcomes: correct patient selection, acquisition, interpretation, synthesis, and then implementation of a therapy with proven benefit, however, still remains an important goal for future research to enhance adoption of ccTEE.^81^

### ccTEE for the Patient in Shock

For the patient in shock, a transthoracic approach is typically used given its availability and non-invasive nature. In many critically ill patients, however, transthoracic windows are limited.^10,42^ For these patients, ccTEE is indicated and may give additional diagnostic yield beyond TTE.^42,43,82^

**Figure 2.**
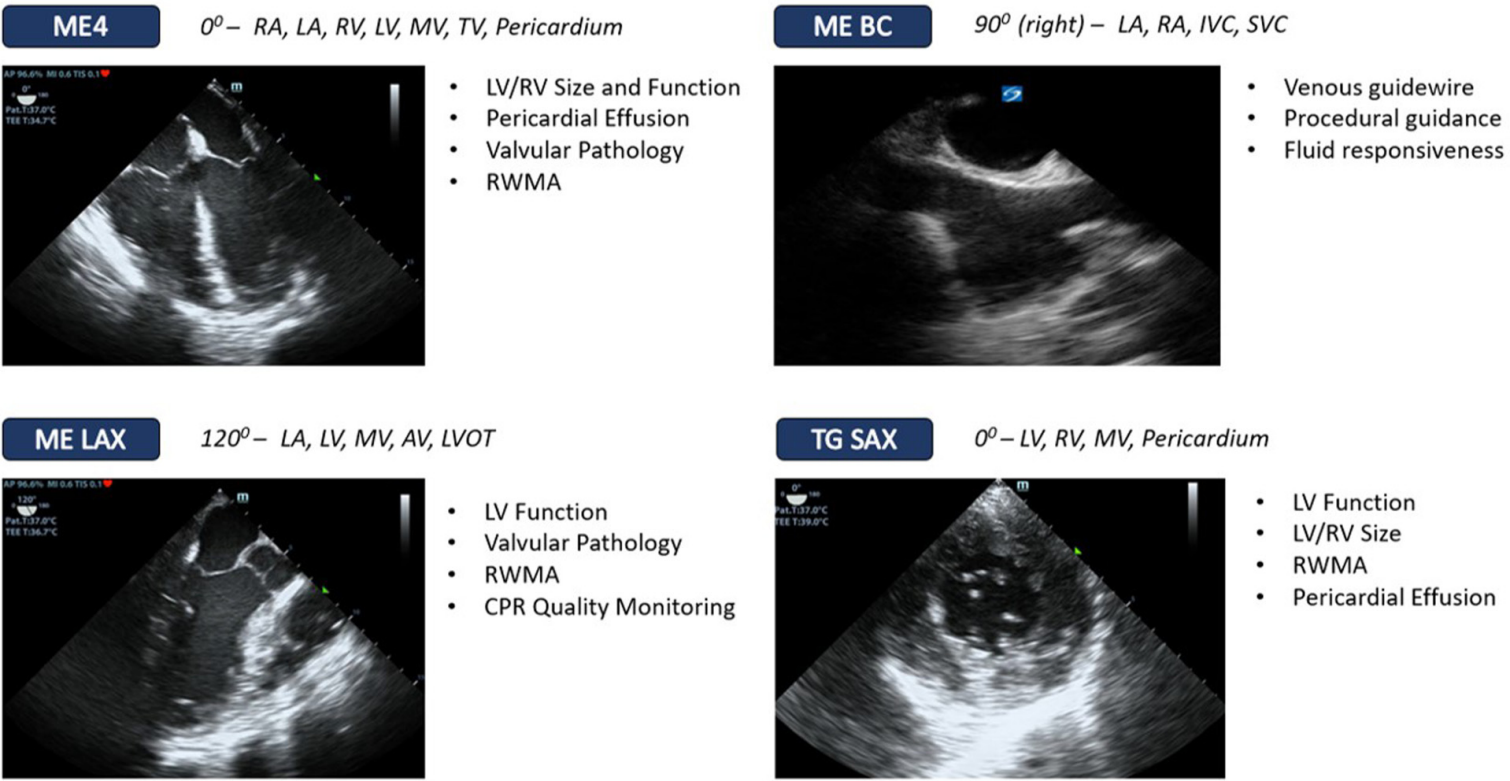
Resuscitative ccTEE views used for phenotyping of shock in the unstable patient Legend. Abbreviations: ME4, Mid-esophageal 4 chamber; MEBC, Mid-esophageal bicaval; MELAX, mid-esophageal long axis; TG SAX, Transgastric short axis; LA, Left Atrium; RA, Right Atrium; MV, Mitral Valve; TV, Tricuspid Valve; AV, aortic valve; LVOT, Left Ventricular Outflow Tract; LV, Left Ventricle; RV, Right Ventricle; RWMA, Regional Wall Motion Abnormalities; CPR, Cardiopulmonary Resuscitation.

Perhaps the most powerful application of ccTEE is its ability to quickly phenotype shock and identify causes that have targeted treatments such as pulmonary embolism, effusion, or severe ventricular dysfunction.^30–40,83,84^ Its use to phenotype shock is valuable even when only core “resuscitative” TEE views are used, although refining management often requires additional views and quantitative measurements. A simplified “resuscitative” ccTEE protocol like the one shown in Figure 2 allows for the assessment of LV and RV function, pericardial disease, and catastrophic valvular failure.^23^ Given the scalable complexity of ccTEE, these core views can be safely and effectively performed by intensivists with only focused training.^85^

After phenotyping shock, ccTEE can play an important role in refining its ongoing management through titration of fluids, inotropes, and vasoactive medications. One approach to fluid resuscitation is to restrict IV fluids to patients with proven fluid responsiveness with signs of end organ hypoperfusion. Transthoracic approaches to predicting fluid responsiveness have variable diagnostic accuracy and generalizability, in part due to requirements for patients to be ventilated, not spontaneously breathing, and in sinus rhythm.^44,45,86–89^ ccTEE measured SVC respiratory variability is an attractive approach to assessing fluid responsiveness given its relative ease of acquisition, repeatability, and moderate predictive accuracy, however, additional prospective studies are needed to properly define positivity thresholds.^44–46^

For patients with ventricular failure, ccTEE is valuable to provide serial assessments of biventricular function during the titration of inotropes, as well as non-invasive cardiac output assessment using LVOT velocity time integral measurements (VTI). Although thermodilution derived cardiac output measurements from pulmonary artery catheters have similar accuracy to TEE,^90^ ccTEE provides the advantage of offering anatomic information that may also inform the mechanism behind any distorted cardiac output physiology.

Given the diagnostic potential of ccTEE for the management of shock, there are a number of areas for future research. Prospective studies assessing ccTEE markers of fluid responsiveness are needed to determine the diagnostic accuracy and thresholds for test positivity. Ultimately, however, studies assessing the impact on patient important outcomes from integrating ccTEE into resuscitation paradigms are needed to help justify more widespread adoption of the technique.

### ccTEE use During Cardiac Arrest

POCUS use in cardiac arrest has been adopted by many clinicians, particularly for the identification of reversible causes and futility of further resuscitation.^91,92^ Despite its enthusiastic implementation, some studies suggests POCUS TTE is associated with longer pulse checks, which is antithetical to high quality CPR.^11,93^ TEE affords reliable acoustic windows to guide clinicians without interfering with chest compressions or prolonging pulse checks.^11^ This can help identify arrythmias like ventricular fibrillation not seen on ECG (Video 3),^32,36,52^ and also distinguish true pulseless electrical activity (PEA) from pseudo-PEA, with the latter having higher survival rates and the potential to respond to pharmacologic therapies.^52,94^

The use of ccTEE in cardiac arrest also has the potential to improve CPR quality. The American Heart Association suggests to perform compressions mid chest on the lower portion of the sternum compressing to a depth of at least 5cm,^95^ however, from observational TEE data this results in almost half of chest compressions occurring over the LVOT or aortic root. CPR over the aortic root in animal and human studies is ineffective, with little cardiac output generated and poor survival.^13,52,96^ ccTEE can be used to reposition compressions over the LV in real-time (Videos 1 & 2).^52^

There are several areas for future research for intra-arrest TEE. These include the feasibility of implementing intra-arrest TEE to improve CPR quality and whether this correlates with improved proximal (end-tidal CO_2_ and arterial pressure) and distal outcomes (ROSC and survival). Additionally, as more intra-arrest TEE is performed and pseudo-PEA is detected, determining the optimal therapeutic management of this entity is needed.

### Procedural use of ccTEE

ccTEE also has a role for the initiation and management of patients on ECMO. In some centers, ECMO cannulation is done with fluoroscopic guidance, however, TEE guided cannulation is an alternate strategy that may increase its portability.^55,58^

For centers performing ECMO, the ability to troubleshoot flow problems and catheter placement is essential. TEE can help identify catheter misplacement, recirculation of venous blood, and mechanical complications from the cannula. Additionally, the weaning of VA-ECMO requires serial assessments of ventricular function which is feasible by intensivists and provides flexibility with respect to the timing of weaning trials, however, the optimal strategy for VA-ECMO weaning is still an area of important research.^60^

### ccTEE use in Trauma

In trauma, the extended focused assessment with sonography in trauma (eFAST) exam has become a well integrated into acute resuscitation. The role of ccTEE in trauma patients is poorly defined, however, it has been shown to be feasible with some diagnostic value in select clinical cases.^63–65,97^ Regional mediastinal or pericardial hemorrhage in particular, typically occult to TTE, can be readily identified on TEE.^17,41^ Additionally, when there are no transabdominal or transthoracic windows due to subcutaneous emphysema or body habitus, ccTEE can potentially sequence immediate priorities or disposition. The “TREE” protocol for acute trauma resuscitation TEE focuses on identifying hypovolemia, aortic injuries, pericardial disease, and pleural pathology,^98^ however, prospective studies validating its use are lacking.

Given the relative paucity of studies assessing ccTEE use in trauma, prospective research assessing the feasibility of integrating ccTEE into the management of major trauma is warranted to ensure that it offers additional diagnostic information to existing trauma algorithms, and that it does not delay the sequencing of other life saving interventions or disposition.

### ccTEE use for Respiratory Failure

A natural extension to TEE use for shock has been for the evaluation of patients with severe respiratory failure. These patients may have acute cor pulmonale and intracardiac shunting that can be identified with ccTEE.^74–76,99,100^ Identifying these pathologies may help optimize ventilation strategies, PEEP, and the use of inhaled pulmonary vasodilators.

Additionally, transesophageal lung ultrasound (TELUS) is highly effective at imaging the posterior and basal lung zones, which often contain pathology for the intubated critically ill patient (Video 4).^73^ TELUS can detect significant consolidation that may account for clinically important hypoxia, even when it is not visible on chest x-ray.^73^ Additional research to assess the diagnostic accuracy of TELUS and also the impact of TEE guided lung recruitments is needed (Video 5 & 6).

### Safety of ccTEE

Probe insertion is a commonly cited concern for clinicians learning ccTEE. The complication rate of ccTEE in the literature is low, with no major complications such as death or perforation reported, although there is likely underreporting due to the retrospective nature and ad-hoc reporting of studies. Gastrointestinal bleeding is a known risk of TEE, however, most cases are self limited and do not require intervention.^78^ As well, incomplete reporting of whether patients were intubated limits the ability to generalize complication rates to different ICU populations. Additional prospective research systematically screening for complications is important to ensure that clinicians can accurately weigh the risks and benefits of ccTEE.

### Limitations

There are several limitations to this study. Incomplete reporting of primary TEE studies, particularly with respect to who performed the TEE, made it difficult to assess whether the TEE was consultative or goal-directed. In these cases, discussion between authors was used to generate consensus using other domains like the setting (eg, ED or pre-hospital) and indication (eg, cardiac arrest) to help determine whether the TEE was comprehensive or ccTEE. Given this and the scope of this review, there is likely incomplete retrieval of the ccTEE literature. As well, ad-hoc reporting of complications in many studies means that the true complication rate is still unknown and is an important area for future research.

### Conclusions

Although critical care TEE is still in its relative infancy, the indications and clinician-reported impact of ccTEE have been preliminarily characterized. Additional studies evaluating the impact of ccTEE on patient outcomes are still forthcoming and will assuredly have the greatest impact on priority domains for ccTEE adoption.

## Abbreviations

ccTEE: Critical care transesophageal echocardiography
TTE: Transthoracic echocardiography
POCUS: Point-of-care ultrasound
CCE: Critical care echocardiography
ICU: Intensive care unit
PRISMA-SCR: Preferred Reporting Items for Systematic Reviews and Meta-analyses extension for scoping reviews
OSF: Open Science Framework
ED: Emergency Department
LV: Left ventricle
RV: Right ventricle
LVOT: Left ventricular outflow tract
IVC: Inferior vena cava
SVC: Superior vena cava
ITT: Intention-to-treat
SHR: Sub-hazard ratio
CI: Confidence interval
VF: Ventricular fibrillation
CPR: Cardiopulmonary resuscitation
PEA: Pulseless electrical activity
VV-ECMO: Veno-Veno extracorporeal membrane oxygenation
VA-ECMO: Veno-Arterial extracorporeal membrane oxygenation
TELUS: Transesophageal lung ultrasound
VTI: Velocity time integrals
AMC: Area of maximal compression
eFAST: Extended Focused Assessment with Sonography in Trauma
ROSC: Return of Spontaneous Circulation

## Appendix

**Appendix 1.** Search strategy.

**Database: Embase Classic + Embase <1947 to 2021 March 31>, Ovid MEDLINE(R) ALL <1946 to March 31, 2021>, EBM Reviews - Cochrane Central Register of Controlled Trials <February 2021>**	
**Search Strategy:**	
1	Echocardiography, Transesophageal/ (69227)
2	(transesophageal or transoesophageal).tw,kf. (54664)
3	((transesophag* or transoesoph* or esophag* or oesophag*) adj2 echo*).tw,kf. (50431)
4	or/1-3 (91021)
5	Intensive Care Units/ or Burn Units/ or Coronary Care Units/ or Recovery Room/ or Respiratory Care Units/ (260831)
6	Critical Care/ (160920)
7	Critical Illness/ (64909)
8	(critical* adj2 (ill* or care)).tw,kf. (220315)
9	intensive care.tw,kf. (404761)
10	Respiration, Artificial/ (192024)
11	(ventilat* adj5 patient*).tw,kf. (98657)
12	Emergency Service, Hospital/ (80855)
13	(emergency adj2 (room* or department*)).tw,kf. (323238)
14	(icu or recovery room* or Respiratory Care Unit* or Coronary Care Unit* or burn unit*).tw,kf. (230306)
15	Emergency Treatment/ (29138)
16	Emergency Medical Services/ (145426)
17	prehospital.tw,kf. (32125)
18	heart arrest/ or out-of-hospital cardiac arrest/ (121827)
19	(cardiac or heart).tw,kw. adj arrest*.tw,kf. (102665)
20	or/5-19 (1498774)
21	4 and 20 (8241)
22	exp animals/ not humans/ (19277949)
23	(exp infants/ or exp child/) not (exp adults/ or Adolescent/) (3263323)
24	((child* or infant*) not adult*).ti. (2209432)
25	or/22-24 (22719507)
26	21 not 25 (5114)
27	**26 use medall (1907) Medline**
28	*transesophageal echocardiography/ (17456)
29	(transesophageal or transoesophageal).tw. (54077)
30	((transesophag* or transoesoph* or esophag* or oesophag*) adj2 echo*).tw. (49853)
31	or/28-30 (58784)
32	intensive care unit/ or medical intensive care unit/ (232858)
33	exp *intensive care/ (313661)
34	intensive care/ (185056)
35	critical illness/ (64909)
36	critically ill patient/ (50634)
37	(critical* adj2 (ill* or care)).tw. (216433)
38	intensive care.tw. (401104)
39	emergency ward/ (236058)
40	(emergency adj2 (room* or department*)).tw. (322218)
41	(icu or recovery room* or Respiratory Care Unit* or Coronary Care Unit* or burn unit*).tw. (229300)
42	emergency health service/ or emergency medical dispatch/ or hospital emergency service/ (228296)
43	prehospital.tw. (31579)
44	exp *heart arrest/ (76418)
45	((heart or cardiac) adj arrest*).tw. (102390)
46	or/32-45 (1510677)
47	31 and 46 (5147)
48	(child/ or infant/) not (adult/ or aged/) (3172356)
49	(exp animal/ or nonhuman/) not exp human/ (12189088)
50	((child* or infant*) not adult*).ti. (2209432)
51	48 or 49 or 50 (16113811)
52	47 not 51 (4808)
53	conference abstract.pt. (4086595)
54	52 not 53 (3541)
55	**54 use emczd (2035) Embase**
56	Echocardiography, Transesophageal/ (69227)
57	(transesophageal or transoesophageal).tw,kw. (55420)
58	((transesophag* or transoesoph* or esophag* or oesophag*) adj2 echo*).tw,kw. (51257)
59	or/56-58 (91172)
60	Intensive Care Units/ or Burn Units/ or Coronary Care Units/ or Recovery Room/ or Respiratory Care Units/ (260831)
61	Critical Care/ (160920)
62	Critical Illness/ (64909)
63	(critical* adj2 (ill* or care)).tw,kw. (225028)
64	intensive care.tw,kw. (413616)
65	Respiration, Artificial/ (192024)
66	(ventilat* adj5 patient*).tw,kw. (99090)
67	Emergency Service, Hospital/ (80855)
68	(emergency adj2 (room* or department*)).tw,kw. (323665)
69	(icu or recovery room* or Respiratory Care Unit* or Coronary Care Unit* or burn unit*).tw,kw. (232072)
70	Emergency Treatment/ (29138)
71	Emergency Medical Services/ (145426)
72	prehospital.tw,kw. (32682)
73	heart arrest/ or out-of-hospital cardiac arrest/ (121827)
74	((cardiac or heart) adj arrest*).tw,kw. (105182)
75	or/60-74 (1504817)
76	59 and 75 (8308)
77	(exp Infant/ or exp child/) not (exp Adult/ or Adolescent/) (3263323)
78	((child* or infant*) not adult*).ti. (2209432)
79	76 not (77 or 78) (7928)
80	**79 use cctr (156) Cochrane**
81	27 or 55 or 80 (4098)
82	remove duplicates from 81 (2833)
83	**82 use medall (1902) Medline**
84	**82 use emczd (854) Embase**
85	**82 use cctr (77) Cochrane**

## Appendix 2. Search strategy for clinicaltrials.gov and Open Science Framework

**Table:**

Line	Search
1	Transesophageal
2	Transoesophageal
3	TEE
4	1 OR 2 OR 3

## Appendix 3. Search strategy for conference abstracts

**Table:**

**Database: Embase Classic + Embase <1947 to 2021 March 31>**	
**Search Strategy: Conferences**	
1	*transesophageal echocardiography/ (9511)
2	(transesophageal or transoesophageal).tw. (31778)
3	((transesophag* or transoesoph* or esophag* or oesophag*) adj2 echo*).tw. (29603)
4	or/1-3 (34259)
5	intensive care unit/ or medical intensive care unit/ (174648)
6	exp *intensive care/ (277415)
7	intensive care/ (130754)
8	critical illness/ (31292)
9	critically ill patient/ (50634)
10	(critical* adj2 (ill* or care)).tw. (125915)
11	intensive care.tw. (227355)
12	emergency ward/ (160799)
13	(emergency adj2 (room* or department*)).tw. (188650)
14	(icu or recovery room* or Respiratory Care Unit* or Coronary Care Unit* or burn unit*).tw. (141189)
15	emergency health service/ or emergency medical dispatch/ or hospital emergency service/ (111454)
16	prehospital.tw. (17584)
17	or/5-16 (928048)
18	4 and 17 (3294)
19	(child/ or infant/) not (adult/ or aged/) (1740386)
20	(exp animal/ or nonhuman/) not exp human/ (7381807)
21	((child* or infant*) not adult*).ti. (1182126)
22	19 or 20 or 21 (9496387)
23	18 not 22 (3106)
24	Society of Critical Care Medicine.so. (10783)
25	Critical Care Congress of the Society of Critical Care Medicine.so. (8586)
26	Society for Academic Emergency Medicine.so. (8968)
27	saem.so. (8965)
28	(chest and annual meeting).so. (8076)
29	24 or 25 or 26 or 27 or 28 (27831)
30	23 and 29 (91)
31	limit 30 to yr = "2018 -Current” (52)

The 6 major conferences searched were: Society for Critical Care Medicine, World Congress of Intensive and Critical Care, European Society of Intensive Care Medicine, Canadian Critical Care Forum, CHEST annual meeting, and Society for Academic Emergency Medicine. Canadian Critical Care Forum and World Congress of Intensive and Critical Care were not able to be included in the above search and the conference abstract PDFs were screened separately.

## Appendix

**Appendix 4.** Study design, indication, main findings, and complications for TEE case studies and small case series

Author and year	Study Design, Clinician and Level of Training	Indication	# of pts	Main Findings	Complications
Jelic et al, 2017	Case Study, EP, NR	Cardiac Arrest	1	86F presented w/ dyspnea, then went into PEA. TEE performed for diagnosis and management. Findings of large RV; small and hyperdynamic LV; distended noncollapsing SVC; suggestive of PE. TEE used for monitoring CPR quality and prognostication.	None
Saranteas et al, 2010	Case Series, Intensivist, NR	Hemodynamic	2	Case 1 - 36M in MVC who required splenectomy. Day 5 in ICU experienced hemodynamic instability prompting TEE. Dense echogenic material identified on TEE coating the catheter in SVC consistent with thrombus. Case 2 - 30F post-op day 35 cesarian section found to have anti-phospholipid antibody syndrome. Deteriorated in ICU and TEE performed for hemodynamic instability. TEE detected thrombus formation in the SVC associated with a central line.	None
Agrawal et al, 2020	Case Study, Intensivist, NR	Hemodynamic	1	49M admitted to ICU for mixed respiratory failure secondary to pneumonia. On day 7 suffered hypoxic cardiac arrest. Post-ROSC TEE showed large RV, dilated IVC, large clot in main and right pulmonary artery. Diagnosis of PE made. Also found atrial septal aneurysm and right-to-left shunt through PFO.	None
Absalom et al, 1997	Case Series, NR, NR	Trauma	2	Patient 1: 17F TEE performed in ICU following blunt trauma with a new murmur. TEE identified a dissection flap that was surgically repaired. Patient 2: 17M TEE performed given high energy mechanism, which showed a dissection flap that led to transfer and surgical repair	None
Lerner et al, 2019	Case Study, EP, TEE credentialed	Cardiac Arrest	1	82F cardiac arrest. TEE inserted to determine etiology of arrest and to improve CPR quality. Post-ROSC the patient was bradycardic and TEE was used to guide and position of transvenous pacemaker as difficult passing it blindly.	None
Giorgetti et al, 2019	Case Study, EP, NR	Cardiac Arrest	1	47M cardiac arrest. TEE used to rule out reversible causes, then was used to position hands for chest compressions to avoid compression over LVOT. Then TEE was used to confirm guidewire placement for VA-ECMO cannulation.	None
Orihashi et al, 2020	Case Series, NR, NR	Cardiac Arrest	4	Case 1: 60F cardiac arrest. ECMO initiated. TEE failed to show retrograde flow in the aorta which identified a misplaced arterial cannula. Case 2: 40M flatline ECG but TEE showed fine ventricular fibrillation which was successfully defibrillated. Case 3: 60F cardiac arrest. TEE identified PE and also used to guide a transvenous pacemaker into proper position Case 4: 30F following vaginal birth cardiac arrest. TEE did not suggest amniotic fluid embolism. Instead, showed hypovolemia, increasing pericardial effusion, and intrabdominal hemorrhage.	None
Merlin et al, 2019	Case Study, EP, 2 h didactic and 2 h of hands-on	Cardiac Arrest	1	51F cardiac arrest. TEE inserted by pre-hospital physician to help identify cause of arrest. Cardiac motion seen so a small bolus of epinephrine and IV fluids were given and CPR held. ROSC obtained.	None
Rublee et al, 2020	Case Study, EP, NR	Cardiac Arrest	1	59M PEA cardiac arrest. TEE showed moderate pericardial effusion. Pericardiocentesis showed blood. TEE identified type A dissection and patient taken to OR.	None
Horowitz et al, 2021	Case Study, Intensivist, NR	Cardiac Arrest	1	34M COVID with RV failure and subsequent cardiac arrest. TEE showed clot in transit. Used to position compressions over LV.	None
Krishnan et al, 2013	Case Series, NR, NR	Procedural	2	Case 1: 71M inadequate TTE views. TEE used to guide transvenous pacemaker insertion. Case 2: 68M inadequate TTE views. TEE used to guide femoral transvenous pacemaker insertion	None
Cooper et al, 2000	Case Study, NR, NR	Trauma	1	60M polytrauma. TEE on day 2 identified partial dehiscence of left coronary cusp of aortic valve. Subsequent hemodynamic deterioration with identified worsening aortic insufficiency. Taken to OR with complete repair.	None
Fagnoul et al, 2013	Case Study, NR, NR	Hemodynamic	1	92M who suffered cardiac arrest. Post-ROSC was in cardiogenic shock and TEE performed was performed pre intra-aortic balloon pump insertion which identified thrombus, leading to cessation of the procedure.	None
Leuverink et al, 2019	Case Study, NR, NR	Hemodynamic	1	36F with refractory shock post laparoscopic cholecystectomy. TEE performed in ICU showed external compression of right atrium from an intrabdominal hematoma by the liver. Laparotomy resolved hematoma.	None
Mocavero et al, 2012	Case Study, NR, NR	Hemodynamic	1	70M with IVC filter in ICU with shock. TEE showed severe TR and an IVC filter tangled in the tricuspid valve. Taken to OR with successful surgical repair.	None
Poppe et al, 2021	Case Study, NR, NR	Cardiac Arrest	1	40F witnessed cardiac arrest. TEE intra-arrest positioned mechanical CPR device over LV then identified free floating thrombus in right atrium leading to thrombolysis.	None
Lee et al, 2019	Case Study, NR, NR	Cardiac Arrest	1	54M cardiac arrest. TEE initially showed small intimal tear in descending aorta. Post ROSC patient found to have pericardial effusion but no proximal dissection. Felt to be due to direct myocardial bleeding from CPR.	None
Koroneos et al, 2006	Case Study, NR, NR	Cardiac Arrest	1	53M 24 days post intracranial hemorrhage who suffered cardiac arrest in ICU. TEE showed RV dilation with clot in transit. Received thrombolytics with achievement of ROSC.	None
Lam et al, 2016	Case Study, NR, NR	Cardiac Arrest	1	65F polytrauma who had a cardiac arrest on post-admission day 4. TEE demonstrated pneumocardium following needle thoracostomy suggesting potential iatrogenic introduction of air from the procedure.	None
Lau et al, 2012	Case Study, NR, NR	Hemodynamic	1	46M 6 weeks post mitral valve replacement with new onset shock. TTE negative. TEE showed fibrous pericardial effusion with tamponade physiology, with resolved hemodynamics post operation.	None
Hsieh et al, 2003	Case Study, NR, NR	Cardiac Arrest	1	45F suffered PEA arrest in recovery room following laparoscopic vaginal hysterectomy. TEE showed clot in transit. Treated with IV heparin.	None
Awad et al, 2006	Case Study, NR, NR	Hemodynamic	1	68F chest pain and cardiac arrest. Post -ROSC ECG suggestive of inferior STEMI. TEE performed show clot in right atrium suggestive of PE. Patient treated with IV heparin with improvement.	None
Blaivas et al, 2008	Case Series, EP, Experience with ECHO, 2 h of TEE video	Cardiac Arrest	6	Case 1: 35M PEA arrest. TEE showed cardiac motion without palpable pulse. CPR stopped and inopressors up-titrated with ROSC. Case 2: 73F cardiac arrest. ECG showed asystole. TEE showed VF and patient defibrillated. Eventually found to be hyperkalemic and treated with ROSC. Case 3: 73F cardiac arrest. ECG showed asystole, TEE showed VF which was defibrillated with subsequent ROSC. TEE also identified atrial clot going into RV. Case 4: 45M cardiac arrest. ECG asystole, TEE showed VF. successfully defibrillated. Case 5: 37M hx DVT/PE with cardiac arrest. Presumed PE, however, TEE showed a type A dissection. Taken to OR with successful repair and recovery. Case 6: 61F with prolonged intermittent VF and asystolic arrest. TEE showed VF when monitor was asystole. Successful defibrillation with ROSC, however, ultimately died.	None
Osman et al, 2020	Case Series, EP, NR	Trauma	5	Case 1: 88F MVC unstable with hemothorax. TEE showed medial flap along ascending aorta suggestive of ruptured traumatic aortic injury which was confirmed on autopsy. Case 2: 73M with 20 foot fall and chest pain and cardiac tamponade. TEE which showed aortic dissection and patient was taken to OR for repair. Case 3: 55M MVC. Limited TTE views and concern for tamponade. TEE showed blunt aortic injury which was taken to the OR. Case 4: 17M MVC. TTE showed possible intimal flap, however, TEE ruled it out. Case 5: 16M motorcycle injury. TEE used to rule out tamponade but confirm traumatic aortic dissection.	None
Simpson et al, 2006	Case Study, NR, NR	Hemodynamic	1	80F admitted with fevers who had hemodynamic collapse post admission day 3. TEE showed dilated RV, clot in the IVC, RA, and pulmonary HTN consistent with PE. Patient received TPA but unfortunately died.	None
Hulin et al, 2016	Case Series, Intensivist, National Board of Echocardiography Certification	Hemodynamic	6	Multiple cases describing the feasibility of TEE to detect abnormal hepatic vein flows, which could suggestive either local or systemic obstructive patterns (RV dysfunction, abdominal compartment syndrome, clot compressing venous return).	None
Peng et al, 2007	Case Study, NR, NR	Hemodynamic	1	45F undergoing dialysis line insertion with hemodynamic collapse. TEE identified large hemothorax compressing the right atrium. Drained with improvement in hemodynamics.	None
Krishnamoorthy et al, 2011	Case Series, NR, NR	Hemodynamic	1	27F fulminant hepatic failure with shock. TEE left in situ for 72 h. TEE identified a clot at the cavo-atrial junction + RV dilation and thickening. Felt that the patient had chronic micro-embolism leading to RV dysfunction. Treated with anticoagulation with improvement. Ultimately diagnosed with catastrophic anti-phospholipid antibody syndrome.	None
Kelly et al, 2019	Case Study, NR, NR	Cardiac Arrest	1	64M cardiac arrest. TEE used to position mechanical CPR device and confirm guidewire placement for ECMO. TEE identified dissection flap so ECMO not initiated.	None
Nowack et al, 2019	Case Series, Intensivist, NR	Trauma	4	Case 1: 32M gun shot wound to chest, subsequent respiratory failure, TEE used to rationalize giving additional transfusion Case 2: 72M post appendectomy, intubated, TEE used to rationalize additional fluid resuscitation Case 3: 58M MVC TEE used to diagnose hypovolemia and give additional fluids Case 4: 24M spinal cord injury and neurogenic shock, TEE used to diagnose concurrent hypovolemia and guide resuscitation.	None
Wei et al, 2010	Case Study, NR, NR	Cardiac Arrest	1	36F post partum day 2 following c-section, TEE showed possible thrombus in pulmonary artery which was confirmed on 3 dimensional TEE. This led to initiation of VA ECMO and pulmonary thrombectomy with survival with good function.	None
Frietman et al, 2001	Case Study, NR, NR	Trauma	1	32M skydiving accident. Aortic arch rupture. TEE initially did not show any clear cardiac abnormality and could not visualize aorta. Patient placed on VV-ECMO for hypoxia. Day 4, TEE repeated showing papillary muscle rupture which was not seen on initial TEE. Underwent successful repair of arch and mitral valve.	None
Wagner et al, 2011	Case Study, NR, NR	Hemodynamic	1	73F with cardiac arrest. Miniaturized hemodynamic TEE post ROSC used to guide resuscitation and showed improvement in cardiac function from post ROSC hour 2 to hour 9.	None
Tsai et al, 2009	Case Study, NR, NR	Cardiac Arrest	1	58F post op day 10 radical hysterectomy with cardiac arrest. TEE showed massive clot in RA which was dislodged through suctioning through a central venous cannula and shaking the patient. The patient regained ROSC and went for ECMO then thrombectomy with discharge and good neurologic outcome.	None
Denault et al, 2003	Case Study, NR, American Board of Anesthesia	Hemodynamic	1	65F post single lung transplant with shock and hypoxia. TEE showed intermittent compression of the right atrium and RV outflow track, ultimately, in keeping with thoracic tamponade secondary to a non-compliant lung post-transplant. TEE then successfully used to guide ECMO cannulation and to wean from ECMO.	None
Turnage et al, 1993	Case Study, NR, NR	Hemodynamic	1	62F with CHF and shock. TTE limited views. TEE found dynamic LV outflow tract obstruction which improved with Esmolol.	None
Cavallaro et al, 2010	Case Study, NR, NR	Hemodynamic	1	77F retroperitoneal hematoma with hypotension, TEE showed systolic anterior motion of the mitral valve with severe subaortic obstruction.	None
Mazzeffi et al, 2013	Case Study, NR, NR	Procedural	1	53F with ARDS and ECMO placement. Poor flows with TEE identifying ECMO cannula in the RV which was repositioned with improvement in flows.	None
Marchandot et al, 2018	Case Study, NR, NR	Cardiac Arrest	1	84F on warfarin for Afib who had a cardiac arrest while undergoing TEE. After ROSC, a left atrial intramural hematoma was noted. This was stable on repeat imaging and didn't require intervention.	Yes
Poularas et al, 2009	Case Study, NR, NR	Trauma	1	26M polytrauma with sup-capsular splenic hematoma. Not well visualized on transabdominal imaging, but able to be monitored for stability with TEE.	None
Liu et al, 2019	Case Series, NR, NR	Cardiac Arrest	4	Case1: 45M out-of-hospital cardiac arrest. TEE used to guide compression over LV Case 2: 84F cardiac arrest, mechanical CPR over aorta which was repositioned with TEE Case3: 54M COPD cardiac arrest. TEE identified compressions over LVOT which were repositioned. Case 4:74F with cardiac arrest, TEE identified compressions of inadequate depth and that they were over the LVOT. Repositioned hand placement with ROSC.	None
Evrard et al, 2020	Case Series, NR, NR	Hemodynamic	5	It is feasible to perform TEE for monitoring hemodynamics of prone patients with COVID19. Of the 5 cases, there were no hemodynamic changes in 3; In one case, severe MR and SAAM was detected.	None

Abbreviations: EP, Emergency Physician; NR, Not reported; PEA, Pulseless Electrical Activity; RV, Right Ventricle; LV, Left Ventricle; SVC, Superior Vena Cava: MVC, Motor Vehicle Collision; ROSC, Return of Spontaneous Circulation; VA-ECMO, Veno-arterial extracorporeal membrane oxygenation; IVC, Inferior Vena Cava.

## Appendix

**Appendix 5.** List of all included studies and case reports

First Author	Year	Journal	PMID
Feissel	2001	Chest	11243970
Liu	2020	Prehospital emergency care	31150302
Chenzbraun	1994	Clinical cardiology	7955591
Poularas	2009	Anesthesia and intensive care	19775061
Tsubo	2004	Critical care medicine	14707563
Fletcher	2015	Journal of cardiothoracic and vascular anesthesia	25575411
Cioccari	2013	Critical care (London, England)	23786797
Varriale	1997	Critical care medicine	9377888
MekontsoDessap	2011	Intensive care medicine	21203747
Krivec	1997	Chest	9367474
Cavarocchi	2013	The Journal of thoracic and cardiovascular surgery	23993027
Mazzeffi	2013	Journal of Cardiothoracic and Vascular Anesthesia	23849533
Mentec	1995	Critical care medicine	7600826
Cavallaro	2010	Minerva anestesiologica	20661209
Vignon	1998	Chest	9631780
Vignon	1994	Chest	7988209
Si	2020	Annals of translational medicine	32647710
Turnage	1993	Chest	8222846
Evrard	2020	Journal of the American Society of Echocardiography	32561110
Bagate	2020	Critical care (London, England)	33287866
Beyls	2020	Journal of the American Society of Echocardiography	32561109
Younan	2019	The American surgeon	31043196
Ruiz-Bailen	2006	Resuscitation	17005313
Marchandot	2018	Heart & lung: the journal of critical care	29506763
Comess	2000	The American journal of medicine	11020390
Held	2016	Journal of burn care & research	26594864
Frietman	2001	Journal of Cardiothoracic and Vascular Anesthesia	11254845
Wei	2011	European journal of echocardiography	21044982
Kelly	2019	The American journal of emergency medicine	30862393
Krishnamoorthy	2011	ICU Director	NA
Lau	2019	Chest	30543807
Nowack	2019	The journal of trauma and acute care surgery	31260428
Peng	2007	Acta anaesthesiologica Taiwanica	17972622
Boissier	2015	Annals of intensive care	25859416
Brederlau	2006	Der Anaesthesist	16900346
Arntfield	2020	Journal of intensive care medicine	30189783
Hulin	2016	A & A case reports	26556108
Huttemann	2002	Intensive care medicine	12185429
Simpson	2006	Critical care and resuscitation	16536716
Osman	2020	The Journal of emergency medicine	32591302
Arntfield	2016	The Journal of emergency medicine	26508495
Awad	2006	Clinical Intensive Care	NA
vanderWouw	1997	Journal of the American College of Cardiology	9283540
Vignon	2017	American journal of respiratory and critical care medicine	27653798
Burrage	2015	International journal of obstetric anesthesia	25683381
Benjamin	1998	Journal of cardiothoracic and vascular anesthesia	9509350
Begot	2015	Intensive care medicine	26254013
Blaivas	2008	Resuscitation	18486300
Merlin	2020	Prehospital emergency care	30957698
Orihashi	2020	Circulation journal	32188835
Griffee	2020	Journal of cardiothoracic and vascular anesthesia	31812567
Wray	2021	Journal of intensive care medicine	31741420
Fair	2019	Annals of emergency medicine	30773413
Denault	2003	Journal of the American Society of Echocardiography	12778031
Lerner	2020	The American journal of emergency medicine	31870671
Giorgetti	2020	Journal of clinical ultrasound	31820822
Younan	2018	American journal of surgery	29439775
Jung	2020	Resuscitation	32653570
Wagner	2011	Resuscitation	21177014
Tsai	1999	Anesthesia and analgesia	10589616
Absalom	1997	Heart	9391296
Agrawal	2020	Annals of the American Thoracic Society	31891302
Metaxa	2011	Journal of critical care	21255968
Schneider	2008	Ultraschall in der Medizin	19241511
Donker	2010	Netherlands Journal of Critical Care	NA
Hsieh	2003	Journal of clinical anesthesia	14698370
Nowak-Machen	2016	Perfusion	27125828
Evans	2014	BMJ case reports	24879737
Verma	2009	Hospital practice	20877173
Hwang	2009	Academic emergency medicine	19732038
Burns	2005	Journal of Trauma - Injury, Infection and Critical Care	16096536
Chimot	2014	Journal of ultrasound in medicine	25425371
Garcia	2017	Chest	28694197
Colreavy	2002	Critical care medicine	12006793
Sapp	2020	The American surgeon	32223796
Teran	2019	Resuscitation	30779977
Sohn	1995	Mayo Clinic proceedings	7564542
Koroneos	2007	Resuscitation	17084012
Lee	2019	Acute and critical care	31723934
Catena	2019	Resuscitation	30825552
Smith	1995	The New England journal of medicine	7823997
Vieillard-Baron	2006	Intensive care medicine	16855828
Lam	2016	Journal of Medical Ultrasound	NA
Slama	1996	Intensive care medicine	8905426
Vieillard-Baron	2013	Intensive care medicine	23287876
Charbonneau	2014	Critical care (London, England)	25189403
Etherington	2010	Journal of burn care & research	20061835
Merz	2019	Intensive care medicine	31273416
Lau	2012	International Journal of Perioperative Ultrasound and Applied Technologies	NA
Saranteas	2010	Anesthesia and intensive care	20514974
Jelic	2017	The Journal of emergency medicine	29128035
Ecker	2013	The New England journal of medicine	23534575
Fagnoul	2013	Revista Brasileira de terapia intensiva	24553517
Griffin	2017	The American surgeon	28822391
Cooper	2000	Critical care and resuscitation	16597297
Vignon	1995	Circulation	7586266
Orme	2009	British journal of anesthesia	19151420
Leuverink	2019	Netherlands Journal of Critical Care	NA
Denault	2002	Canadian journal of anesthesia	11861348
Rublee	2020	Chest	32386652
Poppe	2021	BMJ case reports	NA
Hlaing	2018	Journal of cardiothoracic and vascular anesthesia	29174659
Mocavero	2012	HSR proceedings in intensive care & cardiovascular anesthesia	23440548
Fair	2016	The American journal of emergency medicine	27318746
Goeddel	2019	Anesthesia and Analgesia	31425202
Krishnan	2014	Journal of Cardiothoracic and Vascular Anesthesia	24011910
McLean	1998	Anesthesia and intensive care	9513664
Kim	2021	The American journal of emergency medicine	31982225
Horowitz	2021	CASE	33495743

## Appendix 6. Additional MEDLINE search for studies assessing ccTEE in ARDS and respiratory failure

**Table:**

**Ovid MEDLINE(R) ALL <1946 to March 29, 2022>**	
**Search Strategy:**	
1	Echocardiography, Transesophageal/ (22272)
2	(transesophageal or transoesophageal).tw. (22005)
3	((transesophag* or transoesoph* or esophag* or oesophag*) adj2 echo*).tw. (20010)
4	1 or 2 or 3 (32988)
5	intensive care unit/ or medical intensive care unit/ (65054)
6	exp *intensive care/ (37708)
7	Critical Care/ (57779)
8	Critical Illness/ (35321)
9	(critical* adj2 (ill* or care)).tw. (88488)
10	intensive care.tw. (168971)
11	emergency ward/ (81230)
12	(emergency adj2 (room* or department*)).tw. (132119)
13	(icu or recovery room* or Respiratory Care Unit* or Coronary Care Unit* or burn unit*).tw. (80473)
14	emergency health service/ or emergency medical dispatch/ or hospital emergency service/ (123967)
15	prehospital.tw. (13786)
16	Respiratory Distress Syndrome/ (22686)
17	ards.tw. (15888)
18	acute respiratory distress syndrome.tw. (18683)
19	respiratory failure.tw. (36312)
20	exp Pneumonia/ (246064)
21	exp Respiratory Insufficiency/ (66380)
22	exp Pulmonary Edema/ (17656)
23	Thoracic Injuries/ or Rib Fractures/ (15795)
24	Pneumothorax/ (17796)
25	exp Pulmonary Atelectasis/ (6900)
26	Lung Diseases, Obstructive/ or Pulmonary Disease, Chronic Obstructive/ (64393)
27	5 or 6 or 7 or 8 or 9 or 10 or 11 or 12 or 13 or 14 or 15 (480601)
28	4 and 27 (1606)
29	16 or 17 or 18 or 19 or 20 or 21 or 22 or 23 or 24 or 25 or 26 (462280)
30	**28 and 29 (155)**

## Appendix

**Appendix 7.** TEE for respiratory failure

Study	Study Type and Design	Clinician, Location, and Training	Primary Outcome(s)	Patients (n)	Main Finding(s)	Compli-cations
Boissier et al 2015	Prospective Observational	Intensivist, ICU, Board Certified	Association between presence of TPBT and outcomes	216	A moderate to large TPBT was found in 26% of patients with ARDS and was associated with a hyperdynamic state but did not influence oxygenation. It was associated with higher mortality and less ventilator free days.	None
Dessap et al 2015	Prospective Observational	NR, ICU, “Trained Operators”	Mortality	752	The prevalence of acute cor pulmonale (ACP) during moderate to severe ARDS is 21.8%. Patients with severe ACP had higher mortality (57% vs. 42%, *P* = .03).	None
Legras et al 2015	Prospective Observational	NR, ICU, NR	Prevalence of ACP	195	For patients with moderate to severe ARDS, the prevalence of ACP was 24.6% in this study. The prevalence of PFO was 14.9%.	None
Lheritier et al 2013	Prospective Observational	Intensivist, ICU, “Experience with CCE”	Prevalence of ACP and PFO	201	For patients with ARDS, prevalence of ACP was 22.5%. The prevalence of PFO was 15.5%, with only 12.9% of those with PFO having moderate and consistent shunting. The remainder had small or intermittent shunting. There were no large shunts	None
Dessap et al 2011	Prospective Observational	NR, ICU, NR	Feasibility of Prone TEE	34	TEE was feasible to insert in the prone position in all patients. Its insertion resulted in a change in management in 70% of cases	None
Dessap et al 2010	Prospective Observational	NR, ICU, NR	Prevalence of shunting PFO	203	For patients with ARDS, moderate to large PFO shunting was detected in 19.2% of patients.	None
Tsubo et al 2004	Prospective Observational	NR, ICU, NR	Change in lung density on TEE post proning	10	Assessing left lower lung densities in patients who were proned for ARDS. The density area decreased and PaO2/ FIO2 improved after prone position in ten patients (*P* < .01)	None
Vieillard-Baron et al 2001	Prospective Observational	NR, ICU, NR	Prevalence of ACP	75	For patients with ARDS, 25.3% of patients developed ACP. ACP was associated with longer mechanical ventilation and the need for prone positioning.	None

Abbreviations: TPBT, Transpulmonary bubble transit; ARDS, Acute respiratory distress syndrome; ACP, Acute cor pulmonale.
